# Behavioral Implications of the Covid-19 Process for Autism Spectrum Disorder, and Individuals' Comprehension of and Reactions to the Pandemic Conditions

**DOI:** 10.3389/fpsyt.2020.561882

**Published:** 2020-11-16

**Authors:** Tuba Mutluer, Ceymi Doenyas, Herdem Aslan Genc

**Affiliations:** ^1^Department of Psychiatry, School of Medicine, Koç University, Istanbul, Turkey; ^2^Research Center for Translational Medicine, Koç University, Istanbul, Turkey; ^3^Department of Psychiatry, Koç University Hospital, Istanbul, Turkey

**Keywords:** anxiety, autism spectrum disorder (ASD), behavior, pandemic (COVID-19), parents, psychopathology

## Abstract

During disasters and pandemics, vulnerable populations such as patients with mental conditions are known to be overly influenced. Yet, not much is known about how the individuals with autism spectrum disorder (ASD), one of the most common neurodevelopmental conditions globally with a prevalence of 1%, are affected from health-related disasters, especially the current Covid-19 pandemic. Therefore, we conducted an investigation of how individuals with ASD responded to Covid-19 in terms of comprehension and adherence to implemented measures; changes in their behavioral problems; and how their caregivers' anxiety levels relate with these behavioral changes. Our sample consisted of 87 individuals with ASD (15 girls; ages ranged from 3–29, with an average of 13.96 ± 6.1). The majority of our sample had problems understanding what Covid-19 is and the measures it requires. They also had challenges in implementing social distance and hygiene-related regulations of the pandemic. The majority stopped receiving special education during this period. We observed a Covid-19-related clinical presentation that resembled PTSD in individuals with ASD in terms of increased stereotypies, aggression, hypersensitivity, behavioral problems, and sleep and appetite alterations. All subscales of Aberrant Behavior Checklist (ABC) differed significantly between before and after the pandemic conditions. The number of hours the children slept significantly decreased from before to during Covid-19. The anxiety levels of caregivers were high and correlated with the current behavioral problem levels of their children, but not with the level of their behavioral problems before the pandemic. The difference in ABC total score and specifically the lethargy/social withdrawal subscale score predicted parents' anxiety score. Our results suggest that the Covid-19 period inflicts specific challenges to individuals with ASD and their caregivers, underlining the need for targeted, distance special education interventions and other support services for this population.

## Introduction

Disasters and plagues, such as Covid-19, impact individuals with severe and chronic mental conditions disproportionately (1). Yet, there seems to be scant evidence on how individuals with Autism Spectrum Disorder (ASD), one of the most common neurodevelopmental conditions worldwide, react to disaster conditions. With the numerous disasters worldwide of terrorist attacks, tsunamis, hurricanes, bombings, and earthquakes, the lack of focus on their psychological implications for children with ASD started coming to attention (2). Yet, beyond one study showing individuals with ASD to have decreased adaptive behaviors after exposure to an earthquake, and another indicating disaster awareness training to increase disaster preparedness of children with ASD, there is no data on how individuals with ASD react to disasters in general (3, 4). Additionally, beyond any previous disasters, there is no information on how individuals with ASD are affected by pandemics. Specifically, the repercussions of the global lifestyle changes induced by the Covid-19 pandemic on the autism population are unknown. Given their specific profiles of social interaction difficulties, restricted and repetitive behaviors, and having special education as the only validated intervention, individuals with ASD likely face difficulties over and beyond those experienced by the general population, and other psychological, physiological, and social implications for this population, which is the focus of the current investigation.

Pandemics are similar to other disasters in their unpredictability, fatalities, and persistent effects, yet, they are dissociate from disasters as they prevent victims from converging and gathering and instead requiring the opposite reactions of separation, isolation, and quarantine, which end up interfering with family norms and rituals that generally protect family functioning during crises (5). Such rituals are especially relevant to the ASD population, where repetitive behaviors and interests are a defining feature of the condition and affected individuals adhere to rigid daily rituals. Beyond their health and fatality consequences, pandemics of infectious diseases tend to induce widespread anxiety and psychological problems (6). The current pandemic, Covid-19, has been declared a public health emergency of international concern by the World Health Organization (7). A recent study found the symptoms of children with attention deficit hyperactivity disorder (ADHD) to significantly worsen during the Covid-19 outbreak and emphasized the need to focus on special vulnerable populations during the pandemic (8). Though no such study exists for individuals with ASD during Covid-19, the Covid-19-related risk factors Zhang et al. speculated for children with ADHD seem applicable to individuals with ASD as well, especially the loss of daily routine, inability to access and receive care from primary care settings, and the increased worry of parents further exacerbating children's psychological well-being and increasing their behavioral problems.

ASD is characterized by disturbances in social communication with limited and repetitive areas of interest, which start in early childhood and usually remain lifelong (9). According to the most recent incidence rate, it affects 1 in 54 individuals (10). Most patients also have comorbid intellectual disability and other psychiatric conditions such as ADHD, anxiety disorders, disruptive/impulse-control/conduct disorders, depressive disorders, and obsessive-compulsive disorder (11). Developmental pediatrician Sharon Smile considers children and youth with ASD to be “vulnerable to the effects of prolonged isolation and quarantine and may have difficulty adapting to this new form, especially as inflexibility and insistence on sameness are hallmark characteristics of this disorder” and highlights the need for easily implemented programs that address the needs of children with ASD and their families (12). One such program was described in Italy, where after parents of children with ASD reported their children no longer being satisfied with their usual reinforcers, becoming increasingly uncooperative, and displaying high stereotypy and problem behavior levels, researchers formulated a protocol through their observations and discussions with parents. Verbally interactive children received tutoring and the parents of preschool-aged and minimally verbal children received parent coaching (13). As they did not yet analyze their data, the effectiveness or mediators thereof are not known. Nonetheless, this paper sheds some light on the potential challenges induced by the current pandemic conditions on the ASD population and an initial intervention attempted to alleviate some of these challenges. Another relevant paper is an editorial that presents 10 tips to help parents and caregivers of young children with ASD during the Covid-19 stay at home period (14).

Not much is known about the effects of the Covid-19-related changes created in living conditions on individuals with an ASD diagnosis. Our clinical experiences and the feedback we have received from patients and their caregivers indicate the presence of particular adversities experienced during the pandemic by this population. These adversities can be classified in four subgroups. Firstly, individuals with ASD seem to have a different understanding and knowledge of Covid-19 compared to their peers. This can be explained by differences in abstract thinking, as understanding the non-visible and non-concrete concept of Covid-19, the comprehension of its potential health threats including death, and related reasoning requires abstract thinking (15). Therefore, individuals with ASD may be unable to understand, follow, and benefit from basic preventive methods that are formulated for the general population, which may be inapplicable or challenging for this group. Examples we observed are particular challenges with social distancing and tolerating long durations in home-isolation. This is corroborated in the recent article written by a researcher with ASD who highlights the importance of support for individuals with ASD to cope with the uncertainties and anxiety of Covid-19 while noting the absence thereof of both social and professional support due to social distancing measures, and the potential mental health consequences of reduced access to their already minimal support networks (16). Secondly, individuals with ASD are strictly bound to daily routines, and the isolation process can disturb them by changing their routine, such as going to school or special education at a specific time. Thirdly, symptoms and behavioral disturbances of individuals with ASD can be expected to increase because of the interruption of intensive behavioral and educational interventions that are effective in creating positive change in these domains (17). This adversity is expected to gain importance as the pandemic is predicted to continue for some time. Interruptions in behavioral and educational interventions may increase the ASD symptoms and behavior problems of individuals with ASD. Lastly, caregivers of children with ASD experience significantly higher levels of stress and anxiety compared to caregivers of typically developing children (18). Social support is shown to be a protective factor against stress in parents of children with ASD, where support from friends emerged as the most important factor, and support from significant others and family were less potent protectors (19). This social support from friends that carries such importance for stress protection in caregivers in children with ASD likely becomes less available during the social isolation measures of Covid-19. Moreover, parents of children with ASD were found to display higher cortisol response to psychosocial stress compared to parents of typically developing children and this increased physiological reactivity to acute psychosocial stress (18) may result in parents of children with ASD experiencing more stress during the Covid-19 pandemic compared to parents of typically developing children, which may be even more aggravated by increased behavioral problems and quarantine-related challenges of their children with ASD. Therefore, parent's stress and anxiety levels and difficulties experienced by the children including but not limited to behavioral problems may reciprocally exacerbate each other over the course of the pandemic.

On the other hand, some Covid-19-related situations may be dealt with easier by some children with ASD and their families. The higher adherence to rules and routines and aversion of socialization and physical contact in individuals with ASD may facilitate following mandated hygiene measures such as frequent hand washing or avoiding physical contact with people or surfaces. The risk of sensory overload may be lowered as the children will be out of home less frequently due to home confinement measures. In their recent qualitative study on children with special needs, of which the majority has ASD, and their parents, Asbury et al. found that a small proportion of participants reported some positive impacts of the quarantine, such as not experiencing the challenges of daily routines as going to school or other public places or anxiety of socializing with others (20). Yet, these ASD-related strengths for dealing with Covid-19 measures are likely to be limited and not balance out the precipitated challenges.

Building on our clinical observations along with previous reports and findings, we have conducted one of the earliest studies to the best of our knowledge about the effects of the Covid-19-related life changes on individuals with ASD. We hypothesized that individuals with ASD would have a poor understanding of Covid-19 and related measures. Our second hypothesis was that their ASD symptoms and related behavioral problems, sensory sensitivity, and sleep patterns would have worsened during the pandemic. Our last hypothesis was that the anxiety levels of their caregivers would have increased during the pandemic.

## Method

### Participants

Our sample comprised 87 individuals with ASD from the patient database of the Koc University Hospital, with 72 (83%) males, mean age of the participants 13.96 ± 6.1, and an age range of 3–29 years. The inclusion criterion was having been diagnosed with ASD according to DSM-5 criteria by child psychiatrists with over 10 years of experience in ASD. These patients are regularly followed up in the child psychiatry outpatient unit every 2 months and have up-to-date medical records of their evaluations. The exclusion criterion was having a severe neurological disease or a complex genetic syndrome.

### Procedure

**Figure 1:**
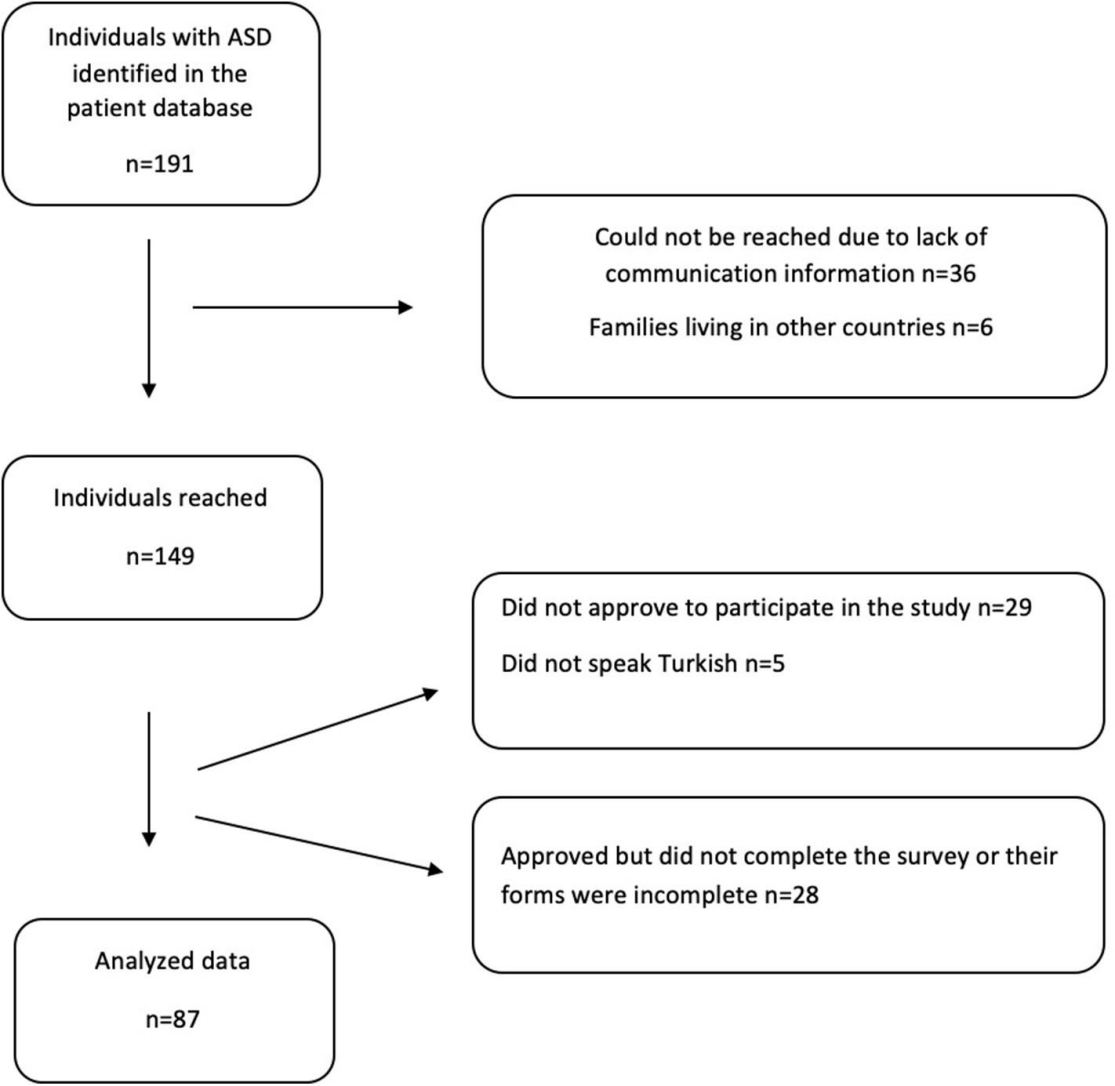
Flow chart of patient recruitment, data collection, and participation rate.

We detected 191 patients diagnosed with ASD in our patient database. The psychologists from our department reached families via phone, introduced the study and invited them to participate. The participation rate was 46% (Figure 1). After parents' verbal assent for the study participation, we sent families an online survey link comprising the written informed consent, sociodemographic form, and the following questions and questionnaires. We questioned the comprehension about Covid-19, communication methods and reactions to pandemic measures of the individuals with ASD. Questions comprised whether tics, stereotypical behaviors or appetite were affected. The sensory hypo/hypersensitivity level, Aberrant Behavior Checklist (ABC), sleep parameters were questioned for before and during the Covid-19 measures. The primary caregivers' anxiety level was assessed by Beck Anxiety Inventory (BAI) for present time only. In addition to the parent-reported surveys, relevant data from medical records were collected.

### Measures

*Sociodemographic form*. The age, gender, educational level of the participant, special education status, number of siblings were collected through a sociodemographic form.

*Clinical registration data*. In order to determine our sample characteristics, we collected ASD-related clinical information from the clinical registration system of our hospital. We rated autism severity according to the level of support level required as defined in DSM-5; language level as the three categorizations of “absence of language,” “speech via words only,” “speech via sentences”; and IQ as indicated through “no intellectual disability (ID) or borderline IQ,” “mild ID,” and “moderate or severe ID.” We recorded other psychiatric comorbidities and assessed their severity according to the Clinical Global Impression scale with ratings ranging between 1 (Normal, not at all ill) and 7 (among the most extremely ill patients).

*Pandemic-related questions*. These questions probed our participants' knowledge and understanding about Covid-19, adaptation to the Covid-19-related measures, special education situation, and access to online resources were assessed through parents addressed questions such as how much their child with autism understands the pandemic, child's level of understanding of the explanations made when he/she wants to go out, reactions to the use of masks, gloves, disinfectants, if they are continuing their education, and if they are using conference or other communication applications or portals during the pandemic.

*Aberrant Behavior Checklist (ABC)*. Changes in ASD-related symptoms and behavioral problems after one and a half months of Covid-19-related measures were assessed by a parent reported aberrant behavior checklist (ABC) for their child for the present and for before the measures for the pandemic started. This data was cross-referenced and otherwise supplemented by the participants' medical records from the hospital, to eliminate any recall bias. ABC is a four-point Likert type scale, which was developed to evaluate the behavioral problems observed in individuals with ASD and intellectual disabilities (21). It has been used to measure the effects of pharmacological, behavioral, and other treatments on these behaviors (22, 23). It contains 58 items that resolve onto five subscales. The subscales and the numbers of items are as follows: (a) irritability (15 items), (b) lethargy/social withdrawal (16 items), (c) stereotypic behavior (7 items), (d) hyperactivity/noncompliance (16 items), and (e) inappropriate speech (4 items). Score for the self-injury factor can be obtained using three items from the irritability factor. Severe self-injury is defined as a total combined score of 3 or greater (24). Turkish validity and reliability study was done by Karabekiroglu and Aman (25).

*Pittsburgh Sleep Quality Index (PSQI)*. PSQI is a 19-item questionnaire for evaluating subjective sleep quality over the previous month (26). We used four items from PSQI to collect information about the sleep quality and disturbances of the participants before and after the pandemic measures. Single items were shown to moderately or highly correlate with PSQI total score and previous studies used single-item sleep measures (26–28). We used four items each representing one of the following components; subjective sleep quality, sleep latency, sleep disturbance and sleep duration within the 1-month during the pandemic measures and 1-month earlier than that period. Turkish validity and reliability study was done by Agargün et al. (29).

*Beck Anxiety Inventory (BAI)*. We measured the level of primary caregivers' anxiety during the pandemic period by administering BAI. BAI is a 4-point Likert type questionnaire measuring the severity of self-reported anxiety. It comprises 21 items scored between 0 (not at all) and 3 (severely). According to the total score of BAI, anxiety level is obtained as minimal (0–7 points), mild (8–15 points), moderate (16–25 points), and severe (26–63 points) (30). The BAI has been reported to be valid and reliable (31).

### Statistical Analysis

Statistical analyses were performed using the software IBM-SPSS Statistics, Version 26.0. Descriptive data were reported as numbers and percentages or as mean (M) ± standard deviation (SD) according to the nature of the data. The continuous variables about before and during the pandemic measures were compared with paired samples *t*-test and multivariate repeated measures ANOVA test. Spearman correlation analyses were made to calculate the association between continuous variables. We conducted a linear regression analysis to determine child-related predictors of parent anxiety.

## Results

Our participants were aged between 3 and 29, with a mean age of 13.96 ± 6.1. They included 15 girls (17%) and 72 boys (81%), paralleling the global male-to-female ratio of ASD. The sociodemographic information about the participants is depicted in Table 1. Our clinical ASD sample had a high rate of comorbidity, where 78% had at least one psychiatric comorbid condition. In this sample, 25% had ADHD, 30% had mood disorders, 15% had tic disorders, 5% had anxiety disorders, and 4% had other comorbidities.

**Table 1:** Sociodemographic information.

	**Mean ± SD (min–max)**	
Child's age	13.96 ± 6.1 (3–29) *n* = 87	
Mother's age	42.32 ± 6.9 (27–60) *n* = 85	
Father's age	46.80 ± 7.3 (28–65) *n* = 87	
Mean severity of child psychiatric comorbidity scores according to CGI (1–7)	2.49 ± 0.1 (1–5) *n* = 87	
	**Frequency (%), number (*****n*****)**	
Education status	Kindergarten	9% (8)
	Elementary school	26% (23)
	Middle school	15% (13)
	High school	22% (19)
	University	2% (2)
	No school	25% (22)
Receiving special education		84% (73)
Number of siblings	Only child	20% (17)
	One sibling	53% (46)
	Two siblings	22% (19)
	Three siblings	6% (5)
Severity of autism (DSM-5 based)	Mild	48% (39)
	Moderate	27% (22)
	Severe	26% (21)
Verbal ability	Can speak with sentences	51% (44)
	Can speak with words	24% (21)
	Cannot speak at all	25% (22)
Intellectual disability	None or borderline	46% (40)
	Mild	36% (31)
	Moderate or Severe	18% (16)
Psychiatric comorbidities	None	22% (17)
	ADHD	25% (22)
	Mood disorders	30% (26)
	Anxiety disorders	5% (4)
	Tic disorders	15% (13)
	Other comorbidities	4% (3)
Medication usage	Overall	76% (66)
	Anti-psychotics	59% (51)
	Antidepressants	13% (11)
	Stimulants or Atomoxetine	17% (15)
	Mood stabilizers	14% (12)
	More than two medications	36% (27)
Medical comorbidities	All medical comorbidities	22% (19)
	Epilepsy comorbidity	14% (12)

Parent-reported information related to their child's Covid-19-related understanding, adaptation to the Covid-19-related requirements, special education situation, and access to online resources can be found in Table 2. Parent responses about behavioral, appetite, sleep, and other problems experienced by their children during the pandemic are given in Table 3. When asked about the changes in their child during the pandemic period, 55% of the parents said that their child got more aggressive, 26% said their child's tics increased or new tics emerged, 29% said their child's communication skills deteriorated, and 44% and 33% of the parents reported sleep and appetite changes, respectively.

**Table 2:** Parent-reported information of Covid-19-related understanding and problems of individuals with ASD.

**Question**	**Response**	**Frequency (%), number (*n*)**
Are there any individuals in your family being treated for Covid-19?	Yes No	2% (2) 98% (85)
Is your child's special education continuing during the Covid-19 process?	Yes No	8% (78) 92% (78)
How well did your child understand what Covid-19 is?	Not much	58% (49)
	Medium	21% (19)
	Well	20% (17)
How well did your child understand Covid-19-related measures and necessities such as staying home and social distancing?	Not much	47% (40)
	Medium	28% (24)
	Well	24% (20)
Is your child able to follow Covid-19 measures (staying at home, keeping social distance etc.)?	Yes No	55% (46) 45% (37)
Can your child perform measures such as washing hands, wearing masks and gloves, and using disinfectants under your instruction?	Yes No	80% (66) 20% (17)
Is your child experiencing sensory problems while implementing these measures of wearing masks and gloves, washing hands, and using disinfectants?	Yes No	37% (31) 63% (52)
Do you use a resource explaining what Covid-19 is and what needs to be done?	Yes	20% (17)
	No	80% (66)
If you are not using it, would you want such a resource for children with ASD, and if it existed, would you use it?	Yes No	85% (56) 15% (10)
Is your child using online, remote conference media such as Zoom, Teams, etc.?	Yes No	29% (24) 71% (60)

**Table 3:** Parent-reported behavioral and other problems during the pandemic.

	**Frequency (%), number (*n*)**
Problems related to ASD	
Communication skills deteriorated	29% (25)
Stereotypies increased	14% (12)
Hypersensitivity increased	14% (12)
Behavioral problems other than ASD	
Aggression	55% (48)
Tics (increased or new tics emerged)	26% (23)
Hyperactivity	56% (49)
Appetite
Increased	12% (10)
Decreased	21% (18)
Sleep
Increased	8% (7)
Decreased	36% (31)
Enuresis Nocturna	1% (1)
Masturbation	2% (2)
Other	51(44)

Our findings show that ASD-related behaviors, sleep quality, and hypersensitivity changed significantly from before the pandemic to during the pandemic. Table 3 presents the comparison of the total scores and subscores of the Aberrant Behavior Checklist (ABC) before and during the Covid-19 measures with paired-samples *t*-test. According to our results, participants showed increased ASD-related behaviors in total and this increase was observed in all subscales, that is, irritability, lethargy/social withdrawal, stereotypical behavior, hyperactivity, and inappropriate speech. We also conducted multivariate repeated measures ANOVA for ABC subscales as the dependent variables at two time points (before and during pandemic) as the within-subject factor. We found that all subscales of ABC differed significantly between the two time points [*F*_(1.0, 83.0)_ = 28.92, *p* < 0.001, η2 = 0.26]. The number of hours the children slept significantly decreased from before to during Covid-19 (Table 4). Participants' hypersensitivity level also increased significantly from before the pandemic to during the pandemic period (Table 4).

**Table 4:** ASD-related behavior, sleep quality, and hypersensitivity differences before the pandemic and during the Covid-19 process.

	**Before the pandemic**	**During the pandemic**	***t***	***P***	**Cohen's *d***
**Variable**	**Mean ± SD**	**Mean ± SD**			
ABC Total Score	48.4 ± 24.6	57.6 ± 27.7			
**ABC subscales**					
Irritability	12.6 ± 8.1	15.4 ± 9.4	5.3	0.000	0.57
Lethargy/social withdrawal	10.0 ± 5.9	11.8 ± 8.2	3.5	0.001	0.37
Stereotypical behavior	6.2 ± 4.1	7.3 ± 4.6	4.5	0.000	0.49
Hyperactivity	15.9 ± 9.2	18.6 ± 10.3	4.9	0.000	0.54
Inappropriate speech	3.3 ± 2.9	3.9 ± 3.4	3.8	0.000	0.41
Self-injury	1.2 ± 1.8	1.4 ± 2.0	2.0	0.054	0.22
**Sleep related problems**					
Sleep latency	2.3 ± 1.2	2.4 ± 1.2	0.7	0.465	0.08
Sleep disturbance	2.5 ± 1.1	2.8 ± 1.2	2.7	0.010	0.26
Sleep duration (hours)	8.2 ± 2.0	7.6 ± 2.4	−3.0	0.004	0.26
Sleep quality	2.1 ± 0.8	2.2 ± 0.8	2.1	0.040	0.27
**Sensory hypersensitivity**	2.7 ± 1.2	2.9 ± 1.2	2.5	0.015	0.17

*ABC, aberrant behavior checklist*.

Beck Anxiety Index measures indicated that 25% of caregivers of individuals with ASD had minimum anxiety, 29% had mild anxiety, 21% had moderate anxiety, and 25% had severe anxiety symptoms during the Covid-19 period. BAI results of parents and the correlation of this anxiety score with their child's behavioral problems according to ABC total score before and during the Covid-19 period is given in Table 5. Parent anxiety did not correlate with the total ABC score of the child before the pandemic and only significantly correlated with the inappropriate subscale of ABC for the situation of the child before the pandemic. Parent anxiety significantly correlated with the child's total ABC score during the pandemic, and with the irritability, hyperactivity, and inappropriate speech subscales. All the significant correlations are positive, indicating an increase in child behavior in the related domain or in the subscale corresponds to an increase in parent anxiety.

**Table 5:** Anxiety scores of parents and their correlation with their child's behavioral problems before and during the Covid-19 period.

**BAI total score**			
**ABC scores**	**Spearman's rho correlation coefficients**	**Sig. (2-tailed)**	***N***
**Before the pandemic**			
Total score	0.112	0.311	84
Irritability	0.058	0.603	84
Lethargy/social withdrawal	−0.046	−0.677	84
Stereotypical behavior	0.044	0.691	84
Hyperactivity	0.126	0.254	84
Inappropriate speech	0.295^**^	0.006	84
Self-injury	−0.069	0.541	84
**During the pandemic**			
Total	0.267^*^	0.014	84
Irritability	0.215^*^	0.049	84
Lethargy/social withdrawal	0.125	0.259	84
Stereotypical behavior	0.140	0.204	84
Hyperactivity	0.220^*^	0.045	84
Inappropriate speech	0.358^**^	0.001	84
Self-injury	0.027	0.808	84

*ABC, aberrant behavior checklist; BAI, beck anxiety index*.

** *Correlation is significant at the 0.01 level (2-tailed)*.

* *Correlation is significant at the 0.05 level (2-tailed)*.

We also created new variables of difference scores by subtracting the previous (before pandemic) from the current (during pandemic) scores of ABC total scale and ABC subscales. Then, we conducted two different linear regression analyses to explore the predictors of current parent anxiety, where one analysis included ABC total difference score and the other ABC subscale difference scored as predictors. We found that the difference in ABC's total score (Beta = 0.21, *p* < 0.05, R2 = 0.09) and ABC's lethargy/social withdrawal subscale score predicted (Beta = 0.67, *p* < 0.05, R2 = 0.15) the total score of the parents' anxiety. Differences in other ABC subscales did not significantly predict parents' anxiety.

## Discussion

In this study, individuals with ASD were found to be influenced from the current Covid-19 pandemic with a significant worsening in their behavior problems, which significantly predicted their caregivers' anxiety.

Findings from typically developing samples show that during the current Covid-19 pandemic, depression and anxiety symptoms were higher in elementary school children than before the pandemic (32). The Covid-19 lockdown was reported to have a considerable negative impact on typically developing children's psychological, social, and physical wellbeing, while some children had mixed emotions as they also felt happy and relaxed spending time with their families during the lockdown (33). Upon exposure to H1N1 and SARS outbreaks, one-third of children and one-quarter of the parents who were exposed to self-isolation or quarantine manifested post-traumatic stress disorder (PTSD). These findings indicate that pandemic measures may be stressful and traumatizing for all children and also their parents (5). When we conducted this study, there were no publications investigating the reactions of individuals with ASD to a pandemic or other health-related disaster. The only relevant finding showed that after an earthquake, individuals with ASD experienced declines in adaptive functioning predominantly in the socialization domain after 6 months and also 1 year upon the traumatic event (3). After we have collected our results and submitted our manuscript, a few studies were published on this topic. In one study, for the Covid-19 period, parents reported increased anxiety and fear in families with ASD, accompanied by reports of increased distress, stress, and low mood (20). Another parent-reported survey revealed increased difficulties in managing daily activities including free time and structured activities, and less than half reported more intense and more frequent behavior problems in their children during the pandemic (34). In another survey, which has not been peer-reviewed yet, parents reported a lack of support, feeling of helplessness, and their greatest concern as the worsening of ASD symptoms due to changed routines and worsening behavior and concern for their child losing their previously acquired skills (35). Our study is the first to report the specific responses of individuals with ASD to the Covid-19-related behavioral measures and the resulting changes under three main domains: Covid-19-related understanding and reactions, behavioral changes during the pandemic, and how they relate to parent anxiety levels.

Firstly, the individuals with ASD were mostly not able to understand what Covid-19 is, to adapt to its measures of social distancing and staying home, and to perform the relevant hygiene requirements. Specific challenges of ASD, like hypersensitivity, could have an impact on the mask wearing status, and this may result in preventing them to take cautions. These findings parallel the parent reports from different countries on how their children with ASD are coping with and reacting to Covid-19 measures. BBC Scotland reporter noted from personal experience how autism makes it extra hard for affected individuals to endure lockdown mainly because of the rigidity in their thinking and a California writer on the Washington Post shared the challenges children with ASD experience when asked to wear masks due to sensory, anxiety-inducing, and smell-related issues (36, 37). Stankovic et al. found that 40% of the children with ASD had difficulties wearing protective masks or gloves (35). Our findings not only align with reported experiences from around the world, but also carry them beyond personal reports to the realm of scientific measurement. Behavioral challenges of ASD such as hyperactivity and fidgetiness may also result in a diminished adaptation to the suggested hygiene procedures due to an impatience to complete or perform them. These issues not only affect the autistic individuals' infection prevention, but also may lead to an increase in the spread of infection and disease in their family and community.

Secondly, we explored the behavioral difficulties experienced by individuals with ASD during the pandemic through the main problematic areas of core ASD symptoms, aberrant behaviors, sleep schedules, and associated problems of aggression, hypersensitivity, tics, appetite, and self-injury. Parent reports indicated deterioration in communication; increases in stereotypes, hypersensitivity, and aggression; appetite changes; and emergence of new tics or increase of existing tics during the Covid-19 period. From the scale measures, the areas that showed significant worsening through the pandemic period were irritability, social withdrawal, stereotypy, hyperactivity, inappropriate speech, self-injury, sleep disturbance, sleep duration, and sleep quality. Parallel with our results, in a recent study from Italy exploring the impact of Covid-19, parents reported more intense (in 35.5% of children) and more frequent (in 41.5% of children) behavior problems in their children with ASD compared to before (34). Similar to our findings, another study from Turkey also found increased sleep disturbances in children with ASD, such as more bedtime resistance, increased delay in falling asleep, and more night wakings compared to before the pandemic (38). Considering that pandemic life changes could be classified as a trauma-like condition, our reported clinical presentation of Covid-19 reactions in individuals with ASD is likely to have shared characteristics with PTSD. This idea is corroborated by a recent review, which showed that behavioral profiles in individuals with ASD upon experiencing trauma are similar to our findings. Exploring specific trauma symptomatology in ASD, they concluded that PTSD symptoms like aggressive behavior, self-injury, concentration and sleep problems are common in ASD after trauma (39). In another clinical ASD sample with high comorbidity rates like ours, 67% of ASD patients fulfilled the criteria for PTSD (40). It is notable that their main findings of increased behavioral problems (e.g., aggression, self-injury), social-communication deterioration, increased stereotypes, increased hyperactivity, and changes in sleep and appetite in the traumatized individuals with ASD highly overlap with our results. These commonalities suggest that Covid-19 may produce a similar clinical presentation to PTSD in individuals with ASD. This is an important point to consider, since depression and PTSD in individuals with ASD is associated with increased risk for suicidal thought and behaviors (41).

In general, increased anxiety and depression incidence rates have been reported in caregivers of ASD patients (42, 43). In line with the current literature, we found high rates of anxiety symptoms in our ASD primary caregiver population. Twenty nine percent of them had mild, 21% had moderate, and 25% had severe anxiety symptoms during the Covid-19 period. We further evaluated the correlation between primary caregivers' BAI anxiety score (current) and ASD patient's ABC total scores (before Covid-19 and during Covid-19). This relationship was significant for during the Covid-19 period but did not reach significance before the pandemic period. Just as PTSD manifesting in parents and children exposed to quarantines during the SARS and H1N1 pandemics, the Covid-19 pandemic period seems to have influenced their parents as well as the ASD patients (5). Stankovic et al. also found that caregivers of children with ASD had negative emotions such as feeling of helplessness and need for support during the Covid-19 period (35). Our findings highlight the need for interventions targeting both individuals with ASD and their caregivers.

The lack of action plans targeted toward ASD individuals and their families is evident and must be addressed so that they can be applied effectively during collective crisis periods. Whereas measures were taken to ensure mainstream education to continue as distance education during the Covid-19 period, the majority of individuals with ASD in this sample were found not to have access to special education that is the only validated intervention for them. Intervention plans enabling children with ASD to continue special education as soon as possible and ASD-specific materials to explain Covid-19 are needed. It is also crucial that collaboration between professionals specializing in ASD and trauma is established to investigate interventions that can effectively address this trauma-related symptomology in ASD.

The main limitations of our study are its relatively small sample size and the phone interview and online survey method of data collection. Parents were asked to fill questionnaires for two time periods; for during the pandemic (during the time Covid-19 measures were implemented in our country, comprising the last 1 month) and before the pandemic (before the measures for Covid-19 started to be implemented in our country). Though we explained the timing referred to by the two assessments in a very clear way by a phone call right before filling the forms, parents' evaluation of previous behavior may have been influenced from current behavior, and such recall bias can be a limitation of the study. Though parent-report data was corroborated with the medical records in our hospital, we were not able to perform structured face-to-face clinical evaluations due to the social isolation measures of the Covid-19 period. Due to social distancing measures preventing face-to-face clinical evaluations, we were not able to make a diagnostic assessment of PTSD. As PTSD was found to be high in studies conducted after previous quarantines such as H1N1 and SARS in typically developing children, future studies can investigate PTSD in children with ASD in relation to Covid-19. Additionally, future investigations can compare the effect of Covid-19 on children with ASD with its effect on other populations, as our study sample did not involve a control or another diagnostic group to compare the findings and evaluate whether the findings are specific to ASD children and families or not. Another limitation of our study could be the high number of comorbid conditions and related medication usage. Since our sample was recruited from the clinical participant base of the hospital, their comorbidity rates and medication use were high. Future studies recruiting community-based samples can overcome this limitation.

Our study portrays how individuals with ASD were affected by the Covid-19 process shortly after the pandemic. Such an understanding is of key importance in planning psychiatric, psychosocial, and educational interventions for them. Determining these aspects will enable the development and prompt implementation of clinical, psychological, and educational service policies geared toward this population, which is of global importance given its 1 in 54 prevalence. Considering that individuals with ASD exhibit similar difficulties at the face of this pandemic internationally, investigations of ASD populations through clinical and academic expertise emerge as an utmost priority during these trying times.

## Data Availability Statement

The raw data supporting the conclusions of this article will be made available by the authors, without undue reservation.

## Ethics Statement

The studies involving human participants were reviewed and approved by Koç University 2020.168.IRB1.036. Written informed consent to participate in this study was provided by the participants' legal guardian/next of kin.

## Author Contributions

TM, CD, and HA were responsible for study design and contributed to data interpretation and article writing. TM and HA performed data collection. TM performed statistical analyses. CD was mainly responsible for study conceptualization and article writing. All authors personally revised and approved the final version of the manuscript.

## Conflict of Interest

The authors declare that the research was conducted in the absence of any commercial or financial relationships that could be construed as a potential conflict of interest.
